# Left Ventricular Segmentation, Warping, and Myocardial Registration for Automated Strain Measurement

**DOI:** 10.1007/s10278-024-01119-5

**Published:** 2024-04-19

**Authors:** Kuan-Chih Huang, Donna Shu-Han Lin, Geng-Shi Jeng, Ting-Tse Lin, Lian-Yu Lin, Chih-Kuo Lee, Lung-Chun Lin

**Affiliations:** 1 Graduate Institute of Clinical Medicine, College of Medicine, National Taiwan University, Taipei, Taiwan; 2 National Taiwan University Hospital, Hsin-Chu branch, Hsinchu, Taiwan; 3 Division of Cardiology, Department of Internal Medicine, Shin Kong Wu Ho-Su Memorial Hospital, Taipei, Taiwan; 4 Institute of Electronics, National Yang Ming Chiao Tung University, Hsinchu, Taiwan; 5 Section of Cardiology, Department of Internal Medicine, National Taiwan University Hospital, Taipei, Taiwan

**Keywords:** Left ventricular global longitudinal strain, Left ventricular myocardial segmentation, Dynamic time warping, Artificial intelligence

## Abstract

The left ventricular global longitudinal strain (LVGLS) is a crucial prognostic indicator. However, inconsistencies in measurements due to the speckle tracking algorithm and manual adjustments have hindered its standardization and democratization. To solve this issue, we proposed a fully automated strain measurement by artificial intelligence-assisted LV segmentation contours. The LV segmentation model was trained from echocardiograms of 368 adults (11,125 frames). We compared the registration-like effects of dynamic time warping (DTW) with speckle tracking on a synthetic echocardiographic dataset in experiment-1. In experiment-2, we enrolled 80 patients to compare the DTW method with commercially available software. In experiment-3, we combined the segmentation model and DTW method to create the artificial intelligence (AI)-DTW method, which was then tested on 40 patients with general LV morphology, 20 with dilated cardiomyopathy (DCMP), and 20 with transthyretin-associated cardiac amyloidosis (ATTR-CA), 20 with severe aortic stenosis (AS), and 20 with severe mitral regurgitation (MR). Experiments-1 and -2 revealed that the DTW method is consistent with dedicated software. In experiment-3, the AI-DTW strain method showed comparable results for general LV morphology (bias − 0.137 ± 0.398%), DCMP (− 0.397 ± 0.607%), ATTR-CA (0.095 ± 0.581%), AS (0.334 ± 0.358%), and MR (0.237 ± 0.490%). Moreover, the strain curves showed a high correlation in their characteristics, with *R*-squared values of 0.8879–0.9452 for those LV morphology in experiment-3. Measuring LVGLS through dynamic warping of segmentation contour is a feasible method compared to traditional tracking techniques. This approach has the potential to decrease the need for manual demarcation and make LVGLS measurements more efficient and user-friendly for daily practice.

**Supplementary Information:**

The online version contains supplementary material available at 10.1007/s10278-024-01119-5.

## Introduction

Left ventricular global longitudinal strain (LVGLS) has become the most useful prognostic predictor in the past 10 years [1]. Numerous echocardiographic studies have been conducted across a wide spectrum of cardiac diseases to strengthen the clinical usefulness of LVGLS over other traditional echocardiographic parameters. Despite the enthusiasm in literature, the application of LVGLS is still limited in routine practice due to time-consuming and measurement variability. Nowadays, the speckle tracking technique remains the predominant method employed for measuring LVGLS [2, 3]. As a result, it is challenging to eliminate discrepancies in strain measurements that arise from vendor-specific speckle textures, the chosen model for elastic registration, and various data regularization algorithms [4, 5]. On the other hand, the advent of point-of-care ultrasound has introduced a variety of new echocardiographic data that currently lack support for strain analysis [6–8]. Therefore, there is an urgent need to develop a strain method with higher feasibility and compatibility.

Unlike speckle tracking algorithms, the segmentation of left ventricular (LV) myocardial contours is less constrained by ultrasound machine vendors [9–11]. Besides, it has also been widely studied in other imaging modalities such as computed tomography and cardiac magnetic resonance (CMR). Recently, an online video instance segmentation model (In Defense of Online Models for Video Instance Segmentation, IDOL) [12] has been proposed to provide favorable temporal consistency for frame-by-frame segmentation contours. Intuitively, contours from frame-by-frame LV segmentation can be employed for examining LV strain; however, in order to apply this method practically, suitable myocardial registration tools for strain analysis are necessary. Dynamic time warping (DTW) is an algorithm that measures similarities between two temporal sequences and has been extensively utilized in fields such as speech and gait recognition [13–15]. In echocardiography, this is also a potential approach to establish the pairing relationship of endocardial and epicardial LV borders, thereby facilitating myocardial registration on LV segmentation contours for strain analysis. 

In this study, we compared the ability of myocardial registration between the DTW algorithm and speckle tracking technique on a synthetic echocardiogram image sequence. Then, we developed a DTW strain method that can concatenate to different outputs of LV segmentation contours and assessed its feasibility in the clinical echocardiograms of various cardiac diseases.

## Materials and Methods

### Study Population

This study utilized two mutually exclusive collections of echocardiographic images for different purposes. The first set of images—designated as the training group (Supplementary Table 1)—included echocardiograms from 318 healthy athletes who participated in the 2017 Fédération Internationale du Sport Universitaire (FISU), 23 patients with transthyretin-associated cardiac amyloidosis (ATTR-CA), and 27 patients with regional wall motion abnormalities (RWMA). A total of 11,125 frames of annotated LV contours from the training group were used to train the AI segmentation models.

The second was a set of images—designated as a study group—on which LV strains were calculated by different methods of interest to compare their performances. These images were collected consecutively from 40 patients with normal LV morphology, 20 patients with dilated cardiomyopathy, 20 patients with RWMA, 20 patients with apical hypertrophic cardiomyopathy (AHM), 20 patients with ATTR-CA, 20 patients with severe aortic stenosis for transcatheter aortic valve replacement, and 20 patients of severe mitral regurgitation for surgical treatment (Table 1). Various diseases were enrolled to ensure that our method is feasible across various LV myocardial morphologies. In addition to the mentioned echocardiograms, we conducted an extended sub-study involving CMR images from 20 patients with normal LV morphology. This step ensures the feasibility of our method across different imaging modalities.

**Table 1:** Study population

Study cohort	Experiment	Age (yrs)	Male	BH (cm)	BW (kg)	LVEF (%)	LVEDD (mm)	LVESD (mm)	LV mass (g)
Cardiac echo									
General LV morphology	2 and 3	57.6 ± 16.1	17/40	163.61 ± 9.92	66.97 ± 15.91	71.96 ± 5.73	47.05 ± 3.84	27.52 ± 3.44	169.76 ± 51.77
Apical hypertrophic cardiomyopathy	2	65.3 ± 14.0	19/20	165.90 ± 5.39	68.48 ± 7.37	74.10 ± 5.38	48.75 ± 3.02	27.65 ± 3.09	266.53 ± 93.80
Regional wall motion abnormality	2	65.3 ± 11.2	16/20	164.43 ± 7.36	66.70 ± 11.62	46.72 ± 10.88	55 ± 5.9	42.60 ± 7.17	240.17 ± 51.72
Dilated cardiomyopathy	3	48.6 ± 11.8	14/20	168 ± 9.96	71.65 ± 17.01	32.71 ± 7.98	62.85 ± 5.64	52.80 ± 6.54	289.12 ± 80.55
ATTR-CA	3	64.7 ± 8.4	13/20	160.57 ± 8.21	52.15 ± 9.03	60.27 ± 10.50	39.25 ± 5.69	26.60 ± 3.97	315.35 ± 73.91
Aortic stenosis	3	74.7 ± 12.9	11/20	160.70 ± 8.76	63.85 ± 10.17	67.23 ± 10.82	49.40 ± 6.16	30.90 ± 7.41	227.36 ± 58.22
Mitral regurgitation	3	55.1 ± 13.6	12/20	164.40 ± 9.66	62.55 ± 12.29	71.26 ± 6.09	54.95 ± 6.36	32.1 ± 4.00	236.56 ± 80.54

							LVEDV (ml)	LVESV (ml)	
CMR									
General LV morphology	2	47.0 ± 19.2	18/20	170.78 ± 6.21	73.72 ± 13.24	44.16 ± 20.14	216.27 ± 117.06	137.40 ± 109.43	

*ATTR-CA* transthyretin-associated cardiac amyloidosis, *CMR* cardiac magnetic resonance, *LV* left ventricular, *LVEDD* left ventricular end-diastolic diameter, *LVEF* left ventricular ejection fraction, *LVESD* left ventricular end-systolic diameter

### DTW Strain Method (Fig. 1)

**Fig. 1:**
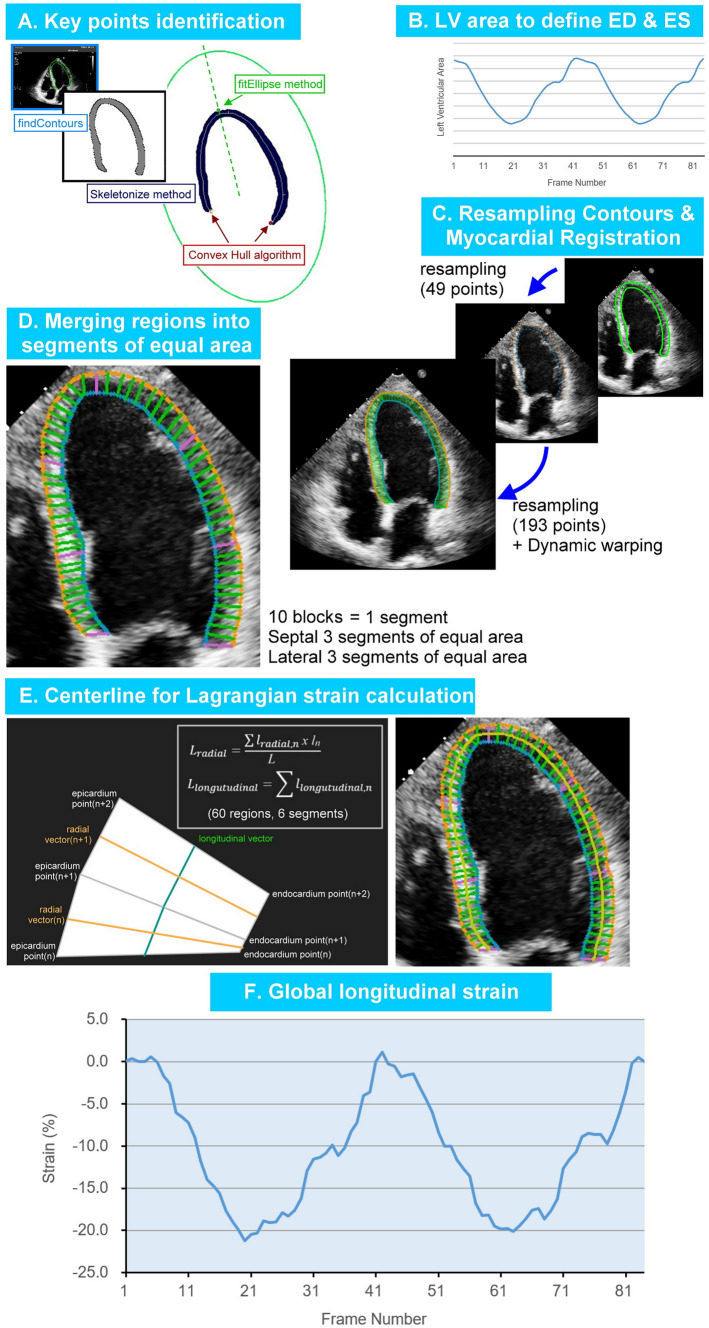
Steps of dynamic time warping (DTW) method of strain calculation. We used the OpenCV findContours program, the skeletonization method, the convex hull program, and the OpenCV fitEllipse program to identify key points including the septal and lateral annular endpoints and the LV apex (A). The calculated LV area defines the end-diastolic and end-systolic frames (B). Key points were used to resample the contour coordinates via interpolation, resulting in 193 epicardial and 193 endocardial coordinates, which were further matched by the DTW method (C). We ensure that the “proportional area” occupied by each block remains constant with respect to that of a corresponding serial number in the end-diastole. The midpoints of each block’s border were connected into the centerline (D). The centerline length of the end-diastolic LV contour is ${l}_{0}$, and the centerline length of frame $i$ is ${l}_{i}$. The $Lagrangian \ strain \ is \ calculated \ as: \frac{{l}_{i}-{l}_{0}}{{l}_{0}}$ (E), and frame-by-frame strain values were represented (F)

Our DTW-based strain calculations include the following steps:

#### Converting Segmentation Border into Coordinates

We used the open-source computer vision library (OpenCV) findContours program to obtain binary coordinates of the LV border. We then used the skeletonization method to reduce the binary image into one pixel-wide representation as a central line with some branches. The convex hull (a program provided by scikit-image) was then used to remove branch points. Since the myocardial contour is U-shaped, if the midpoint of two adjacent coordinates on the convex hull is outside the myocardial contour, these two coordinates should be the septal and lateral endpoints. To identify the LV apical point, we used the OpenCV fitEllipse program to find the ellipse fit of the binary coordinates of the LV contour. We set the long axis of the found ellipse as the *y*’-axis, and the coordinates of the maximal *y*'s value were defined as the LV apical point (Fig. 1A). We defined the end-diastolic and end-systolic frames based on changes in the LV area (Fig. 1B).

The three key points were crucial in defining the septal, lateral, endocardial, and epicardial regions. Subsequently, interpolation was used to resample the endocardial and epicardial contours (Fig. 1C), resulting in 193 points on both the endocardium and epicardium.

#### Myocardial Registration

DTW was utilized to establish correlations between these 193 endocardial and 193 epicardial coordinates, effectively dividing the LV segmentation contour into small triangular/rectangular units. We divided the septal and lateral myocardium into three equal-sized segments for consistency. These smaller DTW units were further merged to form 10 blocks per segment, resulting in a total of 60 blocks in a single frame of LV segmentation contour. These blocks were used as the foundation for myocardial registration.

We assume that there is no out-of-plane motion during ultrasound acquisition, which implies that the total cross-sectional area of the LV segmentation contours should remain constant from frame to frame. Consequently, although the thickness and length of each DTW block may vary with myocardial contraction, the area of each DTW block can also remain constant. This way, we could use the serial order of these blocks to establish temporal correspondence between frames. For this purpose, during the process of combining DTW units into blocks, we maintained proportional area conservation. This ensured that each block occupied an area proportional to its corresponding serial number in the end-diastolic frame, despite slight variations that may arise from the segmentation model. We did not enforce strict area conservation but emphasized proportional area conservation, which was particularly necessary for the septal LV myocardium, whose thickness usually exceeds that of the lateral counterpart. Therefore, we applied the proportional area conservation assumption separately to the septal and lateral segments (Fig. 1D).

#### Strain Calculation

The center points of each block’s border (60 blocks, 61 borders) are connected to form the centerline of the overall segmentation (Fig. 1E). The length of this centerline is used to calculate the Lagrangian strain. The frame of the largest LV area is defined as the end-diastolic frame (Fig. 1B). The centerline length of the end-diastolic frame is defined as *L*_0_. The centerline lengths of the other frames are applied as *L* to the formula (*L* − L_0_) / *L*_0_ to plot the strain curve (Fig. 1F). The maximal absolute value on the strain curve is defined as the peak strain value. In the present study, we tested our proof-of-concept method on apical four-chamber views, and thus the calculated strain values are apical four-chamber view longitudinal strain (A4CLS), to be precise.

### Study Design

There are three experiments in this study to prove the concept of segmentation-based strain measurement.

#### The Experiment-1: “Hopscotch Experiment”

**Fig. 2:**
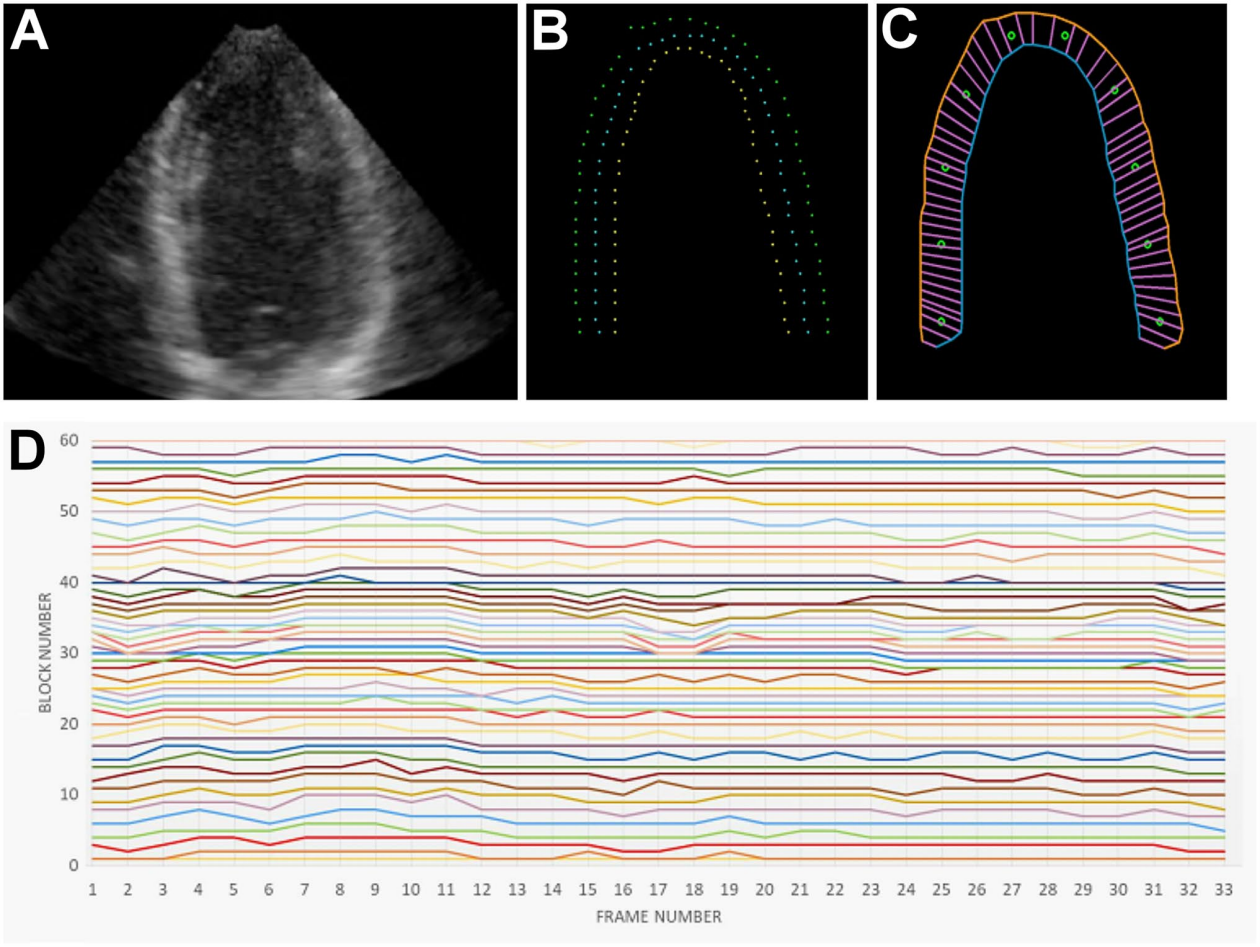
Experiment-1. A simulated apical four-chamber echocardiogram (**A**) is generated to test the registration-like effect of dynamic time warping (DTW). The simulated results of speckle tracking (**B**) were computed independently for the endocardium (yellow dots), mid-myocardium (blue dots), and epicardium (green dots). By overlaying the speckle tracking and the DTW results, the speckle tracking points act as pebbles on the “hopscotch” blocks created by DTW (**C**). The DTW block serial numbers of the 50 mid-myocardial points remain stable within the cardiac cycle of the 33 simulated echocardiographic frames (**D**)

The speckle tracking technique generates multiple “points” along the LV contours, while our DTW method divides the LV contour into blocks. By overlaying these two results, it resembles the classic outdoor game of “hopscotch,” where the speckle tracking points act as pebbles on the blocks created by DTW (Fig. 2C). Each DTW block is assigned with a unique serial number. If the speckle tracking points in different echocardiographic frames have the same DTW block serial number, it indicates that speckle tracking and DTW yield consistent myocardial registration.

We conducted the hopscotch experiment on a synthetic 3D echocardiogram image sequence. A simulated apical four-chamber echocardiography was developed in [16, 17], including ground-truth displacement vectors. Synthetic cardiac data are based on a specific electro/mechanical (E/M) model of the heart driving the positions of a collection of ultrasound scatterers input to the COLE ultrasound simulator [16]. By varying several parameters of the E/M model, different pathophysiological conditions were simulated to produce a loop of 3D ultrasound images over one heart cycle. In the simulated image, three sets of temporally corresponding points (Fig. 2B) were computed using the elastic registration technique for the endocardium, mid-myocardium, and epicardium. Each set consists of 50 points. Since these three sets of points are generated through speckle tracking, they are mutually independent. Because we used the centerline length of the LV myocardial contour to calculate the LV strain, the mid-myocardium tacking points were adopted in the hopscotch experiment.

#### The Experiment-2: Testing of DTW Strain Method

**Fig. 3:**
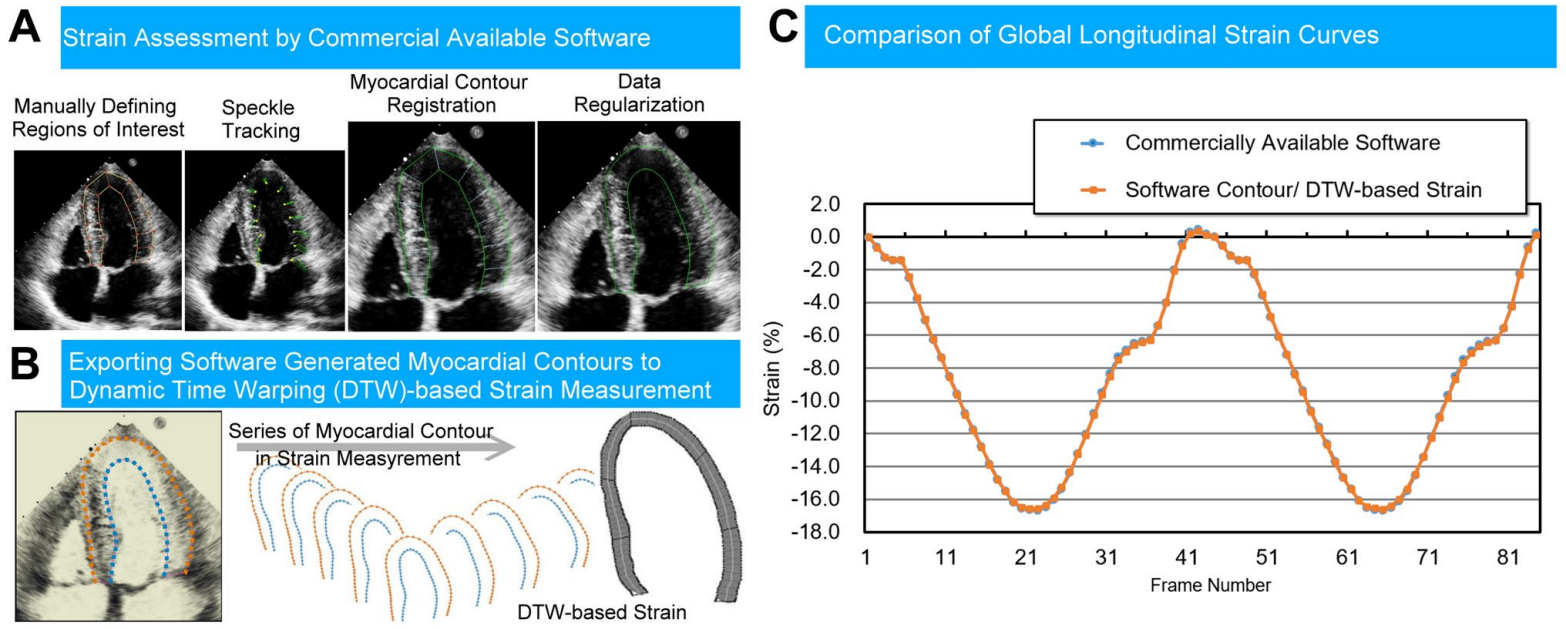
Experiment-2. Manually demarcated LV contours (**A**, left, yellow lines and red dots) are mandatory to start the semi-automated whole-layer LV contour tracking (**A**, middle left, yellow dots and green arrows) and further myocardial registration (**A**, middle right). After data regularization (**A**, right), strain curves were derived from a dedicated software (**C**, blue dots). Frame-by-frame myocardial contours can be exported from the dedicated software (**B**). Dynamic time warping (DTW)-based strain method can provide consistent strain analysis results (**C**, orange dots). These strain curves were highly correlated

The objective of the experiment-2 was to demonstrate the consistency of DTW-based strain calculations with speckle tracking analyses (Fig. 3). Commercially available strain software (TTA 2.3 Cardiac performance analysis, TomTec Imaging Systems, Unterschleissheim, Germany), allowing for manual adjustment of LV borders, was used by an experienced cardiologist in this experiment to demarcate the end-systolic and end-diastolic LV myocardial contours. Subsequently, speckle tracking of the software was used to delineate LV borders in all other frames throughout the cardiac cycle. We performed DTW-based strain calculations on the endocardial and epicardial LV contours exported frame-by-frame. Then we compared these strain values to those generated by the software’s speckle tracking algorithm.

#### The Experiment-3: Combination of Segmentation Model with DTW Strain Method

**Fig. 4:**
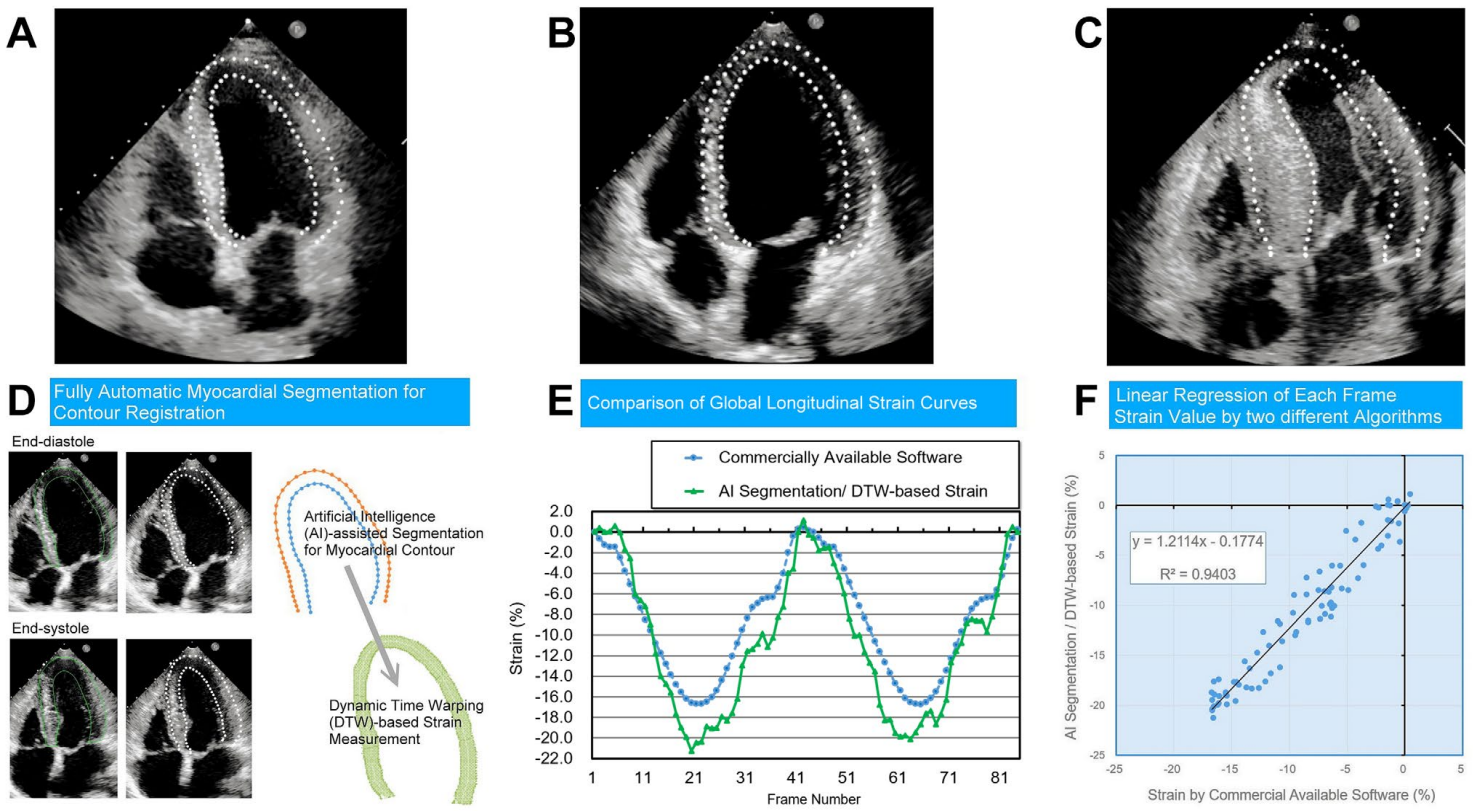
Experiment-3. The online segmentation artificial intelligence (AI) model can provide precise frame-by-frame segmentation for different cardiac pathologies (**A** general morphology, **B** dilated cardiomyopathy, **C** cardiac amyloidosis). Myocardial strain curve calculated by commercially available software for comparison (**E**, blue dots). AI-derived myocardial contours (**D**, left, green lines) were resampled (**D**, middle, white dots) for dynamic time warping (DTW) to calculate myocardial strain (**E**, green dots). These strain curves were linearly well correlated (**F**)

The experiment-3 was conducted after establishing an artificial intelligence (AI) model capable of accurately and consistently segmenting LV myocardium. This AI model was then integrated with the DTW-based strain calculation (validated in the experiment-2) to develop a novel, automated AI-DTW method for strain analysis (Fig. 4). Subsequently, the performance of this AI-DTW method was compared to the same reference method of strain calculation in the experiment-2 of our study.

### AI Segmentation

We trained two AI models for LV contour segmentation: the offline model with Mask-RCNN [18] and the online model with IDOL [12]. The performance metrics of segmentation for the final adapted model were tested (supplementary methods).

### Statistical Analysis

Continuous variables were expressed as mean ± SD and were compared using the paired *t*-test. The Bland–Altman analysis (B-A analysis) and intraclass correlation were used to examine the agreement of peak A4CLS values between different methods. To compare the characteristics of strain curves, we performed a regression analysis of all strain values of each frame between different methods. All statistical analyses were performed using STATA version 13 (StataCorp., College Station, Texas). All reported *p* values were 2-tailed, and *p* values < 0.05 were considered statistically significant.

## Results

### Experiment-1: The Hopscotch Experiment

The LV myocardial contour of the simulated 2D cardiac ultrasound image is divided into 60 DTW blocks. And the mid-myocardium speckle tracking yielded 50 points are served as the pebbles in the hopscotch experiment (Fig. 2C). The block serial number of these 50 points across the 33 frames (1 cardiac cycle) are plotted in Fig. 2D**.** We designated the serial numbers of the DTW blocks where the 50 speckle tracking points were located in the first frame as the reference. For each subsequent frame, if the positions of these points remained the same as in the first frame, the difference should be 0. We calculated the average difference between the positions of these 50 points in the first frame and their positions in the following 32 frames. This served as a quantitative measure for comparing the myocardial registration between DTW and speckle tracking. The average difference in block serial numbers from the first frame is − 0.018. Based on the hopscotch experiment, we can conclude that the DTW myocardial registration aligns consistently with the speckle tracking results.

### Experiment-2: Agreement Between Conventional and DTW-Based LV Strain Calculation Based on Semi-automated Segmentation Contours (Fig. 3 and Table 2)

**Table 2:** Agreement about peak strain values between conventional and DTW-based strain calculation and comparison of strain curves

Experiment-2	LV strain (%)	DTW strain (%)	Bland–Altman analysis		Strain curve regression			
			Bias (%)	LoA (%)	*R*-squared	Beta		
General LV morphology	18.6 ± 3.7	18.6 ± 3.8	0.112 ± 0.047	± 0.590	0.9997	0.9950		
General LV morphology (CMR)	13.0 ± 5.4	12.9 ± 5.4	0.027 ± 0.024	± 0.214	0.9993	0.9935		
Apical hypertrophic cardiomyopathy	16.9 ± 2.9	16.8 ± 2.8	0.072 ± 0.043	± 0.377	0.9984	0.9917		
Regional wall motion abnormality	10.1 ± 3.3	10.8 ± 2.9	− 0.741 ± 0.211	± 1.846	0.9994	0.9989		
Regional wall motion abnormality (regional strain)	11.6 ± 5.1	12.1 ± 5.3	− 0.455 ± 0.208	± 4.463	0.8719	1.0008		

Experiment-3	LV strain (%)	AI-DTW strain (%)	Bland–Altman analysis		Strain curve regression			
					2 loops		Selected beat	
			Bias (%)	LoA (%)	*R*-squared	Beta	*R*-squared	Beta
General LV morphology	19.6 ± 3.5	19.7 ± 3.6	− 0.137 ± 0.398	± 4.935	0.9034	0.9696	0.9422	0.9516
Dilated cardiomyopathy	8.8 ± 2.4	9.8 ± 2.5	− 0.397 ± 0.607	± 5.318	0.8368	0.9234	0.8879	0.9323
ATTR-CA	11.6 ± 3.5	11.5 ± 3.9	0.095 ± 0.581	± 5.096	0.8522	0.9286	0.9175	0.9242
Aortic stenosis	17.4 ± 3.6	17.1 ± 3.0	0.334 ± 0.358	± 3.139	0.8693	0.9893	0.9184	1.0529
Mitral regurgitation	23.2 ± 3.7	22.9 ± 3.5	0.237 ± 0.490	± 4.294	0.9226	0.9706	0.9452	1.0039

*DTW* dynamic time warping, *LV strain* dedicated software-derived LV longitudinal strain from apical four-chamber view, *LoA* limits of agreement

#### Echocardiography with Normal LV Morphology (n = 40)

The B-A analysis of between-method differences of peak A4CLS values revealed a bias of 0.112 ± 0.047% with an estimated LOA (limits of agreement) of ± 0.590%.

The mean *R*-squared value of all these 40 pairs of strain curves is 0.9997 and the mean β value (regression coefficient) is 0.9950.

#### Echocardiography with Apical Hypertrophic Cardiomyopathy (n = 20)

The B-A analysis of between-method differences of peak A4CLS values revealed a bias of 0.072 ± 0.043% with an estimated LOA of ± 0.377%.

The mean *R*-squared value of all these 20 pairs of strain curves is 0.9984, and the mean *β* value is 0.9917.

#### Echocardiography with Regional Wall Motion Abnormalities (n = 20)

Twenty apical echocardiograms of 4-chamber views are used to compare 20 pairs of A4CLS and 120 pairs of segmental longitudinal strain values.

The B-A analysis of between-method differences of peak A4CLS values revealed a bias of − 0.741 ± 0.211% with an estimated LOA of ± 1.846%.

The mean *R*-squared value of all these 20 pairs of strain curves is 0.9994, and the mean *β* value is 0.9989.

The B-A analysis of between-method differences of peak LV regional longitudinal strain values revealed a bias of − 0.455 ± 0.208% with an estimated LOA of ± 4.463%.

The mean *R*-squared value of all these 120 pairs of strain curves is 0.8719, and the mean *β* value is 1.0008.

#### CMR with General LV Morphology (n = 20)

The B-A analysis of between-method differences of peak A4CLS values revealed a bias of 0.027 ± 0.024% with an estimated LOA of ± 0.214%.

The mean *R*-squared value of all these 20 pairs of strain curves is 0.9993, and the mean *β* value is 0.9935.

### Experiment-3: Agreement Between Conventional LV Strain Calculation and Automated AI-DTW Strain Method (Fig. 4 and Table 2)

The pipeline and performance of the AI-DTW method are summarized in Supplementary Table 3.

#### Beat-to-Beat Variation of Strain Curves

In an echocardiographic video consisting of two loops (representing two cardiac cycles), we observed that commercially available software produced identical strain curves for both loops. This means that, upon exporting the coordinates, we discovered that the tracking results were actually identical in both heartbeats within the two-loop video. (We performed regression analysis with the strain value of the 1st and the 2nd loops, and the *R*-squared value is 1.0).

To facilitate a more accurate comparison between our AI-DTW method and the software, we defined the “selected beat” as the first heartbeat within the two-loop video.

#### Effects of Online (IDOL) Versus Offline (Mask-RCNN) Segmentation on Strain Curves

**Fig. 5:**
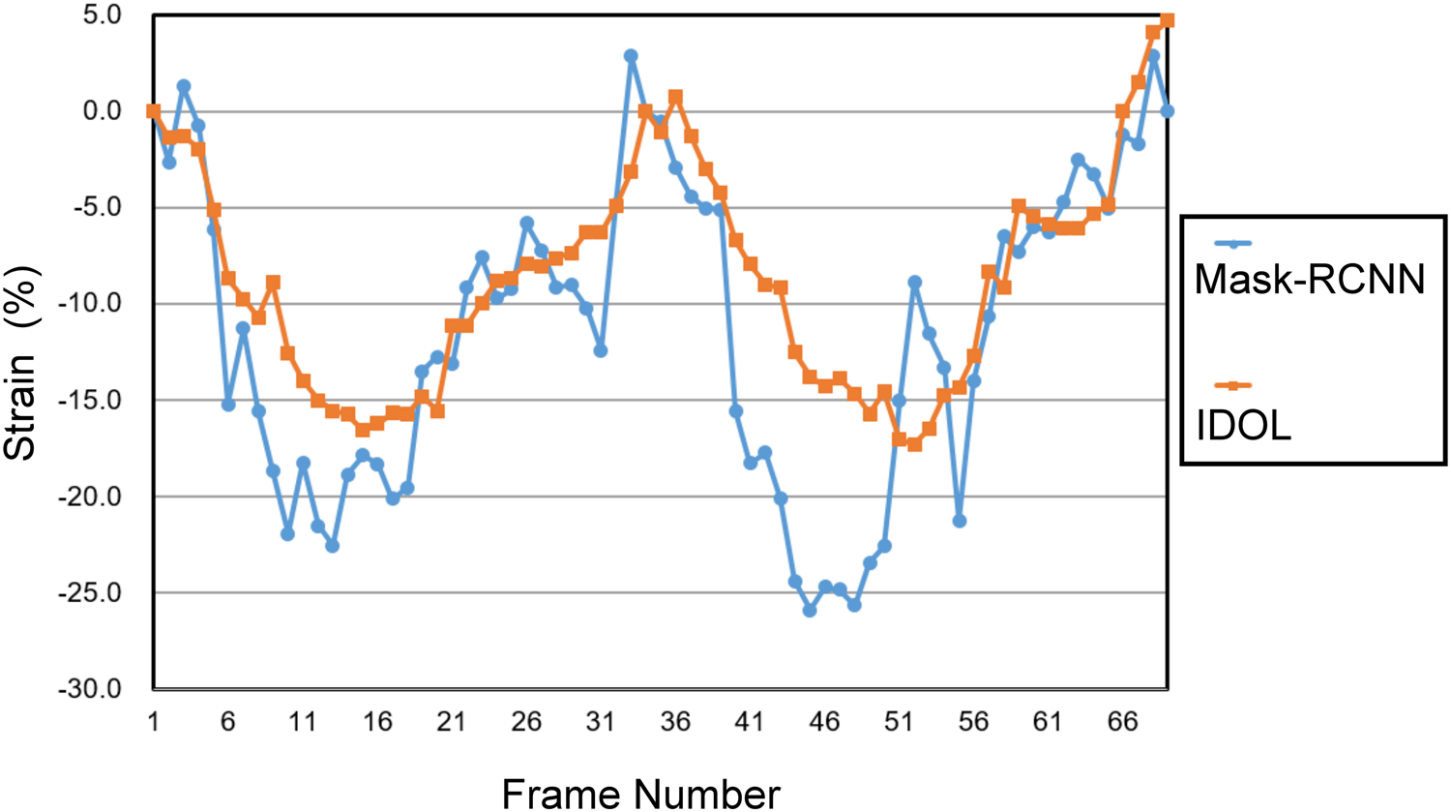
Comparison of the online and offline segmentation yielded strain analysis. The strain curve of the offline segmentation model (blue dots) fluctuated more and could lead to measurement errors in the peak strain values. The online segmentation model (orange dots) was more suitable for segmentation-based strain analysis

We compared the AI-assisted segmentation contour with the semi-automated speckle tracking-derived LV myocardial contour. The Dice coefficient is higher in the selected heartbeat than in the whole video (for the online segmentation model, Dice is 0.805 in the selected heartbeat and 0.745 in the whole 2-beat video, *p* = 0.0007). The Dice coefficient is higher in the online than the offline segmentation model (0.805 vs. 0.709, *p* < 0.0001). Figure 5 demonstrates that the online strain curves exhibit less fluctuations and thus better temporal coherence.

Based on the above findings, in the present study, all of the following AI-DTW strain analysis results were based on the online (IDOL) LV segmentation model.

#### Echocardiography with General LV Morphology (n = 40)

The B-A analysis of between-method differences of peak A4CLS values revealed a bias of − 0.137 ± 0.398% with an estimated LOA of ± 4.935%.

The mean *R*-squared value of all these 40 pairs of strain curve is 0.9034 for the whole video and 0.9422 for the selected heartbeat (*p* < 0.0001); the mean *β* value is 0.9696 for the whole video and 0.9516 for the selected heartbeat.

#### Echocardiography with Dilated Cardiomyopathy (n = 20)

The B-A analysis of between-method differences of peak A4CLS values revealed a bias of − 0.397 ± 0.607% with an estimated LOA of ± 5.318%.

The mean *R*-squared value of all these 20 pairs of strain curve is 0.8368 for the whole video and 0.8879 for the selected heartbeat (*p* = 0.0024); the mean *β* value is 0.9234 for the whole video and 0.9323 for the selected heartbeat.

#### Echocardiography with ATTR-CA (n = 20)

The B-A analysis of between-method differences of peak A4CLS values revealed a bias of 0.095 ± 0.581% with an estimated LOA of ± 5.096%.

The mean *R*-squared value of all these 20 pairs of strain curve is 0.8522 for the whole video and 0.9175 for the selected heartbeat (*p* = 0.0127); the mean *β* value is 0.9286 for the whole video and 0.9242 for the selected heartbeat.

#### Echocardiography with Severe Aortic Stenosis (n = 20)

The B-A analysis of between-method differences of peak A4CLS values revealed a bias of 0.334 ± 0.358% with an estimated LOA of ± 3.139%.

The mean *R*-squared value of all these 20 pairs of strain curve is 0.8693 for the whole video and 0.9184 for the selected heartbeat (*p* = 0.0009); the mean *β* value is 0.9893 for the whole video and 1.0529 for the selected heartbeat.

#### Echocardiography with Severe Mitral Regurgitation (n = 20)

The B-A analysis of between-method differences of peak A4CLS values revealed a bias of 0.237 ± 0.490% with an estimated LOA of ± 4.294%.

The mean *R*-squared value of all these 20 pairs of strain curve is 0.9226 for the whole video and 0.9452 for the selected heartbeat (*p* = 0.1729); the mean *β* value is 0.9706 for the whole video and 1.0039 for the selected heartbeat.

## Discussion

The present study introduces a novel DTW method for myocardial registration that avoids discrepancies caused by tracking algorithms. This method is combined with AI-assisted LV myocardial segmentation results, enabling fully automated strain analysis on a frame-by-frame basis. The *experiment-2* demonstrated the feasibility of our DTW method and the *experiment-3* demonstrated that our AI-DTW strain method is efficient (Table 2) and suitable for various LV morphologies, including DCMP, ATTR-CA, and those with severe valvular heart diseases.

### Segmentation-Based Strain Analysis

The contemporary workflow of LV strain analysis involves several steps, including defining the regions of interest, segmentation, tracking for temporal-spatial registration, and data regularization. Each of these steps can introduce errors in strain measurement [3]. Segmentation is typically the most stable step, as it is less affected by imaging details such as ultrasound speckles. However, performing manual myocardial segmentation frame-by-frame to observe deformation is clinically impractical due to its time-consuming nature.

One possible solution to the problem of detecting endocardial borders is through automation, but currently, it is only available in a limited capacity and still requires manual corrections by experts [19]. To address this challenge, many research pipelines incorporate an AI-assisted segmentation step to define the region of interest for initializing LVGLS measurement [20–22]. However, utilizing various tracking algorithms for subsequent temporal-spatial registration in these specialized AI pipelines may further increase the variability of clinical strain measurements.

Drawing the boundaries of LV myocardial contours manually is a tedious process that demands a lot of effort to learn. Annotation specialists must comprehend the surrounding structures, echocardiography artifacts, and how these elements affect the tracking algorithms. This complexity is further amplified as echocardiography machines advance, providing higher resolutions and frame rates. Moreover, LV strain is primarily used to investigate heart failure with preserved ejection fraction, and a significant portion of this population exhibits varying degrees of myocardial hypertrophy. Increased myocardial thickness will accentuate the variability of tracking algorithms [23]. To achieve accurate segmentation of the left ventricle myocardial contour, assistance from AI-generated technology is necessary.

We have developed a specialized method using DTW to calculate LV strain based on LV segmentation contours. Our method is adaptable to segmentation data series from various techniques. Maintaining temporal consistency is crucial for segmentation models that assess cardiac dynamic functionality [24, 25]. In our study, we found that both mask-RCNN and IDOL performed well in segmenting still frames, with comparable Dice values. However, IDOL was found to be superior to mask-RCNN due to its higher visual continuity. This improvement is reflected in the calculated strain curves (Fig. 5). As AI continues to evolve in cardiac imaging, segmentation remains a crucial process that undergoes constant improvement. Consequently, our method for calculating strain becomes increasingly accurate.

### Strain Curve Characteristics

A group of researchers, led by Sengupta, proposed that analyzing the entire strain curve can be a more precise way of identifying heart diseases [26]. This method is less affected by the variability of strain values compared to solely looking at the peak LVGLS value. Since then, data scientists have used this concept in studying various heart conditions [27, 28]. Combining strain curves with estimated LV pressures has also led to the discovery of myocardial work [29, 30].

However, tracking-based strain analysis has limitations due to accumulation drift, which requires modeling or regularization algorithms for correction. As the number of image frames increases, drift errors accumulate. Consequently, tracking-based strain analysis mainly favors a “selected beat” approach. Additionally, the use of these data processing algorithms can potentially distort the strain curves, introducing excessive variability and compromising the assessment of myocardial function. In contrast, our segmentation-based strain method redefines the end-diastolic frame for each cardiac cycle based on the LV chamber area. This enables us to support strain analysis across multiple beats and enhances the applicability of LVGLS assessment, particularly for patients with atrial fibrillation. In experiment-3, the *R*-squared values of the strain curves generated by both methods were higher for the selected beat compared to 2 loops, further substantiating our hypothesis.

### Cross-Modality and Vendor-Free Strain Analysis

Strain analysis is a crucial aspect of cardiac function evaluation, but different imaging modalities require specific tracking algorithms for accurate results. For instance, speckle tracking is exclusive to echocardiograms, while feature tracking is used for CMR and computed tomography. This could lead to inconsistencies in strain values when comparing results from different imaging modalities.

**Fig. 6:**
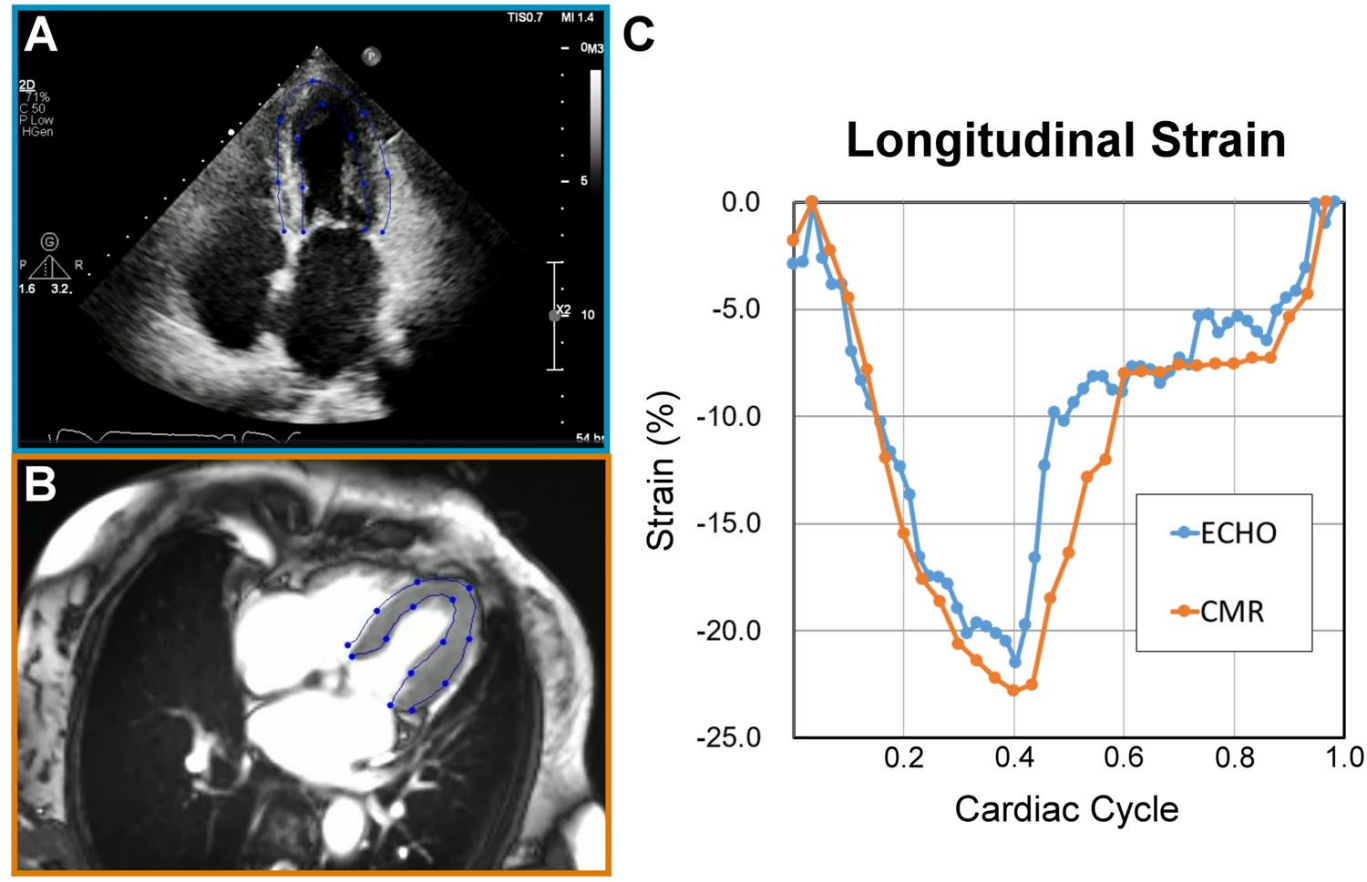
Artificial intelligence-dynamic time warping for cross-modality (echocardiography, cardiac magnetic resonance) strain analysis. The artificial intelligence segmentation model can well demarcate the echocardiography (**A**) and cardiac magnetic resonance (**B**) of the same patient. The dynamic time warping-based method provides comparable strain curves (**C**) for echocardiography (blue) and cardiac magnetic resonance (orange)

In clinical practice, following up on cardiac function often involves different imaging modalities, making it impractical to rely solely on the same vendor’s echo machines. To mitigate this issue, segmentation-based strain analysis provides a vendor-free and cross-modality strain calculation method. Our AI-DTW method uses segmentation contours from the echocardiogram and CMR of the same patient for DTW-based strain calculation, allowing for comparing strain values at different time points even with different imaging modalities. This video-based approach can complete missing strain values in the database, even if DICOM preservation is lacking, as long as the video is available. Figure 6 demonstrates the success of our approach by showing the segmentation contours from the echocardiogram and CMR of the same patient used for strain calculation.

### Study Limitations

While we have proposed and validated the use of LV segmentation contours for conducting LVGLS measurements, our study still has some limitations.

One significant assumption of our DTW method is “proportional area conservation.” This assumption requires that the imaging plane of the echocardiogram passes through the mid-axis of the left ventricle, meaning there should be no foreshortening. This implies a certain level of imaging quality requirement for our method. Common phenomena in echocardiography such as translational motion and out-of-plane motion were not thoroughly examined and validated in this experiment. On the other hand, the number training dataset is small in this proof-of-concept study, a larger and more diverse dataset of echocardiographic images is needed to ameliorate our segmentation model in the future.

While our DTW method has shown promising agreement with conventional techniques in normal LV morphology, DCMP, amyloidosis, and similar pathologies, it still lacks clinical validation in cases with specific left ventricular morphologies such as sigmoidal or reverse curve hypertrophic cardiomyopathy, myocardial infarction-related aneurysm, non-compaction cardiomyopathy, where there are significant variations in left ventricular myocardial thickness. Excessive variations in thickness could potentially affect the results of the DTW method since we rely on DTW to find similarities between the inner and outer edges of the left ventricular myocardium contours to establish registration. Therefore, integrating complementary techniques such as regularization, quality assessment, or post-registration refinement can help alleviate the impact of these limitations and enhance the overall performance of warping algorithms in various applications.

Furthermore, although we have demonstrated that the DTW method can be applied to CMR LV segmentation contours for LVGLS calculation in general LV morphology, we have not yet gathered CMR images for various LV pathologies to develop a dedicated CMR-specific LV segmentation model. Therefore, in cases involving cardiomyopathies, we cannot fully ensure the effectiveness of our method in cross-modality applications. In the future, it is essential for us to collect CMR and computed tomography images for different diseases to validate our approach.

## Conclusion

The online AI model provides LV myocardial segmentation contours with good temporal consistency. Via the registration-like effect of DTW, LV myocardial segmentation contours could be utilized frame-by-frame for the calculation of LVGLS that is consistent with commercially available speckle tracking software. This innovative approach significantly reduces the need for manual delineation, and its cross-modality applicability, vendor-free nature, and video-based features effectively enhance the accessibility of LVGLS assessment in routine clinical practice.

## Supplementary Information

Below is the link to the electronic supplementary material.

Supplementary file1 (PDF 204 KB)

## Data Availability

The data that support the findings of this study are available from the corresponding author, LC Lin, upon reasonable request.
