# Supplementary material for: Left Ventricular Segmentation, Warping, and Myocardial Registration for Automated Strain Measurement
Source: J Imaging Inform Med. 2024 Apr 19;37(5):2274–86. doi: 10.1007/s10278-024-01119-5 (PMC11522271; doi:10.1007/s10278-024-01119-5)
Supplement: Supplementary file 1 — Supplementary file1 (PDF 204 KB) [file 10278_2024_1119_MOESM1_ESM.pdf]

SUPPLEMENTARY MATERIAL

Left Ventricular Segmentation, Warping and Myocardial Registration for Automated Strain Measurement

Supplementary Methods

Off-line model: Mask-RCNN

In the Study Population section, 269 cases served as training sets, while 99 cases were allocated as validation sets for the development of the segmentation model based on Mask-RCNN. The model employed a ResNet backbone and was optimized using stochastic gradient descent with momentum. For regularization, a weight decay of 0.0001 was implemented alongside an initial learning rate of 0.01 and a momentum of 0.9. Training took place on an Nvidia Quadro RTX 6000, utilizing 2 picture-sample per batch and 5000 steps per epoch over 150 epochs. The resulting Dice Coefficient for left ventricular myocardial segmentation achieved by Mask-RCNN was 0.80.

Online model: In Defense of Online Models for Video Instance Segmentation (IDOL)

The IDOL (In Defense of OnLine models) architecture (Supplementary Fig. 1) has revolutionized video instance segmentation by combining advanced deep learning techniques with the DETR (DEtection TRansformer) framework [2]. This has greatly improved the analysis of video sequences for precise instance segmentation. DETR, which is a transformer-based architecture with a DNN (Deep Neural Network) backbone, extracts complex feature maps from images. These feature maps are then processed by a transformer encoder that is enriched with positional encodings. This enhances spatial context and facilitates a deep understanding of the scene through self-attention mechanisms, resulting in image embeddings that encapsulate the complexities of the scene. The transformer decoder further enhances the learnable object queries using these image embeddings, through cross-attention mechanisms, which are crucial for accurate object detection, classification, and segmentation. This effectively targets specific areas within the image. In the final stage, object embeddings undergo multi-head attention to highlight critical features. A Feature Pyramid Network (FPN)-style CNN (Convolutional Neural Network) then transforms these features into precise segmentation masks for each detected object.

The use of contrastive learning in IDOL is crucial for improving the identification of objects in videos. By analyzing the embeddings of video instances and refining them with a contrastive head, IDOL ensures that the representations of the same object in different frames are closer together in the embedding space, while different objects are pushed further apart. This method is advantageous for handling long sequences and ongoing videos, and effectively distinguishes objects across frames. This support of temporal constituency assists our task to segment the left ventricular myocardium in dynamic echocardiography video loops. However, IDOL's reliance on contrastive learning and historical data may create a bias towards situations where objects are easily distinguishable and have minimal interactions, which could limit its performance in cluttered or rapidly changing environments. This bias highlights the challenge of accurately capturing the complexity of dynamic scenes, particularly in busy or varied settings. Therefore, further research is needed to address these challenges and improve video instance segmentation.

The IDOL model was developed with specific hyperparameters to optimize its performance in video instance segmentation tasks. The echocardiographic frame size was 800x600x3 for RGB

images, whereas the model input frame size was configured as 480x360x3 for RGB data. The output mask size was designated as 800x800 to ensure compatibility with subsequent processing steps. The model weight initialization utilized the original IDOL model, which was pre-trained with the YouTube-VIS 2019 dataset, utilizing the SwinL backbone architecture [3]. The loss function employed a multi-task approach, encompassing classification, L1 loss, generalized IoU loss for ROI coordinates, Dice loss and Focal loss for masks, and contrastive loss for discriminative feature embeddings. Regularization was achieved through smooth L1 regularization. The optimization process utilized the Adam optimizer with a learning rate of 0.0001, momentum of 0.9, and weight decay of 0.0001. The IDOL was trained using one video-sample per batch and 256000 steps per epoch for 23 epochs on Nvidia Quadro RTX 6000. **Supplementary Table 1** shows the characteristics of the training population, of which images were acquired with diverse machines, including GE Vingmed Ultrasound Vivid E80, E9, and E95, Philips Medical Systems iE33, and EPIQ 7C.

Evaluation Metrics for the Segmentation Model (IDOL)

Because AI segmentation plays an essential role in the DTW-derived strain measurement, a holistic evaluation of segmentation accuracy was performed in 59 cases (19 athletes, 20 general left ventricular morphology, and 20 transthyretin-associated cardiac amyloidosis), shown in **Supplementary Table 2**.

AI performance

The pipeline from view classification to strain calculation was run on a computer with a GPU card (Nvidia Quadro RTX 6000). The performance for a 2-loop echocardiographic sample video with 101 frames is detailed in **Supplementary Table 3**.

Reference:

1. He K, Gkioxari G, Dollar P, Girshick R: Mask R-CNN. IEEE Trans Pattern Anal Mach Intell. 42:386-397, 2020.
2. Wu JF, Liu QH, Jiang Y, Bai S, Yuille A, Bai X: In Defense of Online Models for Video Instance Segmentation. Available at: https://arxiv.org/abs/2207.10661v1. Accessed 21 July 2022.
3. Wu JF: IDOL model zoo. Available at https://github.com/wjf5203/VNext/blob/main/projects/IDOL/IDOL.md#idol. Accessed 9 Nov, 2022.
4. Chen CJ, Wang YY, Yu JH, Zhou ZY, Shen L, Chen YQ: Tracking pylorus in ultrasonic image sequences with edge-based optical flow. IEEE Trans Med Imaging. 31:843-855, 2012.

Supplementary Table 1 Training Population

	Age	Male	BH (cm)	BW (kg)	LVEF (%)	LVEDD (mm)	LVESD (mm)	LV mass (g)
Healthy volunteer (7456 frames)	46.95±18.56	200/318	163.58±10.67	67.09±13.20	64.94±5.26	48.52±4.64	30.04±4.31	174.89±42.92
Regional wall motion abnormality (2181 frames)	61.93±10.85	20/27	165.67±6.28	72.01±7.63	48.34±10.20	52.56±4.93	39.56±5.92	229.26±55.54
ATTR-CA (1479 frames)	64.63±6.67	14/23	163.65±8.56	52.3±12.19	61.79±7.03	43.57±4.95	29.13±3.33	294.67±105.31

ATTR-CA: transthyretin-associated cardiac amyloidosis, LV: left ventricular, LVEDD: left ventricular end-diastolic diameter, LVEF: left ventricular ejection fraction, LVESD: left ventricular end-systolic diameter.

Supplementary Table 2 Evaluation Metrics for the Segmentation Model (IDOL)

Metrics	By Video (59)	By Frame (2877)
Mask boundary		
Dice coefficient	0.8084 ± 0.0594	0.8155 ± 0.0841
Intersection over Union (IoU)	0.6854 ± 0.0785	0.7004 ± 0.1086
Hausdorff Distance (Pixel)	21.5721 ± 9.1792	20.8882 ± 10.2991
average Hausdorff distance (Pixel) [4]	6.9250 ± 1.7585	7.2001 ± 2.4460
Pixel Class Confidence		
Area under receiver-operating characteristic curves (AUC)	0.9902 ± 0.0074	0.9896 ± 0.1331
Area under Precision-Recall curves (AUPRC), average precision (AP)	0.8658 ± 0.0799	0.8790 ± 0.1071

59 videos in the apical four-chamber view taken from 19 athletes, 20 subjects with general left ventricular morphology, and 20 patients with transthyretin-associated cardiac amyloidosis.

Supplementary Table 3 The Pipeline Performance of Automated AI LV Strain Analysis

Pipeline stage	Input	Output	GPU support	Task	Time per video (ms) (101 frames in this example)	Time per frame (ms)	Note
3D View classifier	DICOM	View tag	N	Preprocess	699	6.92	Filter non-echocardiographic information
			Y	Inference	60	0.59	View classification
IDOL segmentation	DICOM	Mask	N	Preprocess	698	6.91	DICOM converted to PNG
			Y	Inference	263	2.6	Predict mask
Strain calculation	Idol_predicted.pkl	Strain result	N	Preprocess	1320	13.07	Change mask to contour
			N	Inference	1262	12.5	Strain calculation
				Total (ms)	4302	43	

DICOM: Digital Imaging and Communications in Medicine, IDOL: In Defense of Online Models for Video Instance Segmentation, PNG: Portable Network Graphics.

Supplementary Fig. 1 The IDOL (In Defense of OnLine models) architecture

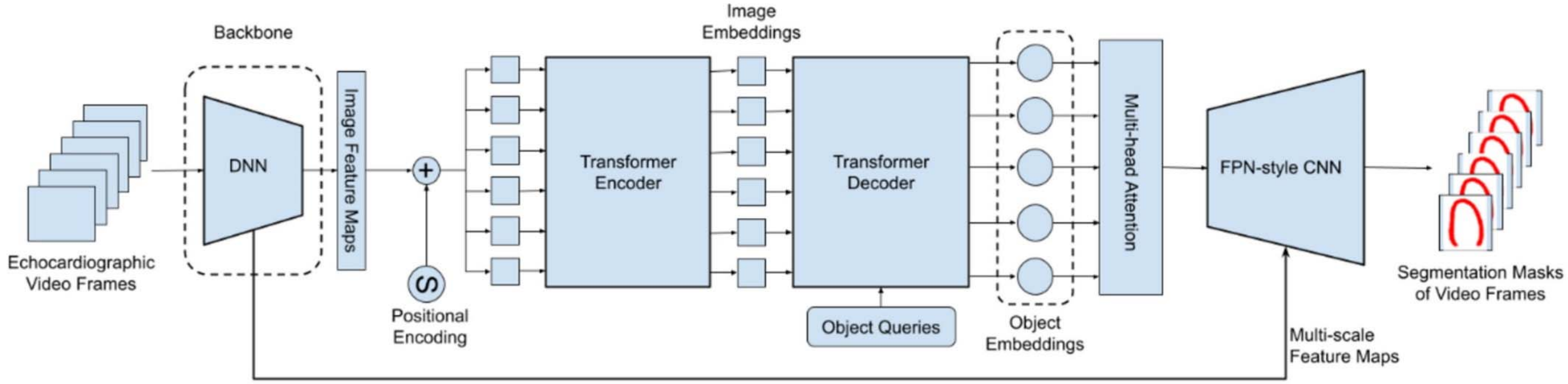

CNN: Convolutional Neural Network, DNN: Deep Neural Network, FPN: Feature Pyramid Network.
